# The Novel Finding of Dynamic Change in eGFR Up to One Year after End of Treatment in HCV-Infected Patients Receiving Sofosbuvir and Velpatasvir

**DOI:** 10.3390/v14020362

**Published:** 2022-02-10

**Authors:** Cheng-Kun Wu, Li-Wei Chen, Te-Sheng Chang, Shui-Yi Tung, Chun-Yen Lin, Chao-Hung Hung, Sheng-Nan Lu, Chih-Lang Lin, Chien-Hung Chen, Chao-Wei Hsu, Tsung-Hui Hu, I-Shyan Sheen

**Affiliations:** 1 Division of Hepato-Gastroenterology, Department of Internal Medicine, Kaohsiung Chang Gung Memorial Hospital, Chang Gung University College of Medicine, Kaohsiung 83301, Taiwan; aasavage.tw@yahoo.com.tw (C.-K.W.); chh4366@yahoo.com.tw (C.-H.H.); juten@ms17.hinet.net (S.-N.L.); e580306@ms31.hinet.net (C.-H.C.); 2 Division of Hepato-Gastroenterology, Department of Internal Medicine, Keelung Chang Gung Memorial Hospital, Chang Gung University College of Medicine, Keelung 20420, Taiwan; leiwei@cgmh.org.tw (L.-W.C.); lion@cgmh.org.tw (C.-L.L.); 3 Division of Hepato-Gastroenterology, Department of Internal Medicine, Chiayi Chang Gung Memorial Hospital, Chang Gung University College of Medicine, Chiayi 613016, Taiwan; cgmh3621@cgmh.org.tw (T.-S.C.); ma1898@cgmh.org.tw (S.-Y.T.); 4 Division of Hepato-Gastroenterology, Department of Internal Medicine, Linkou Chang Gung Memorial Hospital, Chang Gung University College of Medicine, Linkou 333423, Taiwan; chunyenlin@gmail.com (C.-Y.L.); happy95kevin@gmail.com (I.-S.S.)

**Keywords:** direct-acting antivirals, hepatitis C virus, sofosbuvir and velpatasvir, renal function

## Abstract

***Background:*** The results of long-term renal evolution in HCV-infected patients using sofosbuvir and velpatasvir (SOF/VEL), with or without ribavirin (RBV), are lacking. ***Aims:*** We evaluated the renal safety for HCV-infected patients receiving SOF/VEL. ***Methods:*** Between 1 June 2019 and 6 July 2020, we included 594 HCV-infected patients receiving SOF/VEL +/− RBV for 12 weeks in Taiwan. Viral eradication rate (defined by sustained virological response at week 12 post-treatment; SVR12) and changes to renal function were considered. ***Results:*** SVR12 was achieved in 99.3% (590/594) upon per-protocol analysis. Patients saw improved hepatobiliary function and fibrosis after the start of SOF/VEL therapy. For renal function, those with baseline estimated glomerular filtration rate (eGFR) ≥ 60 (mL/min/1.73 m^2^) experienced transient on-treatment reduction in renal function that improved upon ending treatment, but recurrent eGFR degradation during one-year follow-up. The use of RBV (OR = 5.200, 95% CI: 1.983–13.634, *p* = 0.001) was a significant risk factor at SVR24, while diabetes mellitus (OR = 2.765, 95% CI: 1.104–6.922, *p* = 0.030) and the use of RBV (OR = 3.143, 95% CI: 1.047–9.435, *p* = 0.041) were identified as significant risk factors of worsening renal function at SVR48. SOF/VEL did not worsen renal function among those with stage 4–5 chronic kidney disease (CKD) who were not receiving dialysis. ***Conclusions:*** A trend of decline in eGFR at 1 year after SOF/VEL treatment was observed among diabetic patients with baseline eGFR ≥ 60 (mL/min/1.73 m^2^) and concomitant use of RBV. The close monitoring of renal function is warranted. Further study should be conducted in order to weigh the risks and benefit of RBV.

## 1. Introduction

Approximately 71 million patients are infected with the hepatitis C virus (HCV) worldwide [1]. Improvement of quality of life as well as reduction in morbidity and mortality has been observed among those infected with HCV following successful viral eradication. In the era of direct-acting antivirals (DAAs), a high cure rate of >95% of HCV-infected patients has been reported with the direct-acting antivirals sofosbuvir and velpatasvir, in both treatment-naïve and treatment-experienced patients, with interferon (IFN)-based therapy [2,3].

Sofosbuvir/velpatasvir (SOF/VEL) is a pan-genotypic protease inhibitor (PI)-free DAA approved for 12-week treatment in adult patients with or without compensated cirrhosis (Child–Pugh A), and in combination with RBV for those with decompensated cirrhosis (Child–Pugh B or C). This treatment modality boasts a high overall recovery rate of 94–100% reported by clinical trials [4,5,6] across genotype, previous IFN-based treatment history, cirrhosis status, and co-infection. Recently, favorable effectiveness and safety among HCV-infected patients treated with SOF/VEL under normal clinical conditions has also been reported [7,8,9,10].

Renal safety is of concern for SOF-based DAAs. A real-world study (HCV-TARGET) by Saxena et al. reported a higher risk of worsened renal function in patients with a baseline estimated glomerular filtration rate (eGFR) ≤ 45 mL/min/1.73 m^2^, compared to those with eGFR > 45 mL/min/1.73 m^2^ [11]. Since then, some retrospective studies have examined risk associated with on- and off-treatment eGFR, using SOF-based DAAs [12,13]. More recently, Chen-Hua Liu et al. reported on-treatment worsening of eGFR and off-treatment improvement of eGFR in patients receiving SOF-based DAAs [14]. Moreover, a phase II single-arm study demonstrated that treatment with SOF/VEL for 12 weeks was safe and effective in patients with end-stage renal disease (ESRD) undergoing dialysis [15].

Based on these promising results, we aimed to evaluate the real-world efficacy and renal safety of SOF/VEL treatment of HCV-infected patients in Taiwan.

## 2. Material and Methods

### 2.1. Patient Population

Between 1 June 2019 and 6 July 2020, we retrospectively collected data on chronic HCV-infected patients who were treatment-naïve or treatment-experienced for interferon-based treatment at one of four facilities in the Chang Gung Medical Hospital network located in Taiwan (Keelung, Linkou, Chiayi, or Kaohsiung branches). Eligible patients were ≥18 years old and received a sofosbuvir/velpatasvir 400 mg/100 mg tablet once daily for 12 weeks, as well as RBV (adjusted according to body weight) in case of decompensated liver cirrhosis. Patients were excluded if they had a history of human immunodeficiency virus co-infection, solid organ transplantation, or end-stage renal disease under maintenance dialysis. A total of 653 patients were included for intention-to-treat (ITT) analysis. Those lost at follow-up or with absent viral load data at off-treatment week 12 (*n* = 37), cases of mortality (*n* = 21), or chart records not available for review (*n* = 1) were excluded from per-protocol (PP) analysis. Eventually, 594 patients were enrolled for analysis. The study protocol was approved by the ethical committee of the Chang Gung Memorial Hospital (IRB No.: 202100248B0). All patients provided written informed consent.

### 2.2. Study Design

Chronic HCV infection was defined as detectable HCV antibodies (anti-HCV; Abbott HCV EIA 2.0, Abbott Laboratories, Abbott Park, IL, USA) and quantifiable serum HCV RNA (Cobas TaqMan HCV Test v2.0, Roche Diagnostics GmbH, Mannheim, Germany, lower limit of quantification 15 IU/mL) for >6 months. HCV genotype was determined upon screening. The fibrosis stage was defined by transient elastography (FibroScanR; Echosens, Paris, France) and FIB-4 index. Liver cirrhosis was defined by transient elastography with a score > 12.5 kPa, diagnosis by abdominal echography, or the clinical presence of portal hypertension (varices) or decompensation (i.e., ascites, encephalopathy, or gastroesophageal variceal bleeding). Hepatocellular carcinoma (HCC) was defined by cytology, histology, or imaging criteria according to the guidelines of the American Association for the Study of Liver Diseases (AASLD, Alexandria, VA, USA). We collected the baseline demographic and clinical data before the prescription of direct antiviral agents. Patients received laboratory assessment at baseline, on-treatment weeks 4 and 12 (end of treatment; EOT) and off-treatment weeks 12(SVR12), 24(SVR24) and 48(SVR48). Moreover, the eGFR was additionally assessed at on-treatment week 2 by using the isotope dilution mass spectrometry (IDMS) traceable Modification of Diet in Renal Disease (MDRD) equation. The stages of chronic kidney disease (CKD) were defined according to eGFR: 1 = normal (eGFR > 90 mL/min); 2 = mild CKD (eGFR 60–89 mL/min); 3 = moderate CKD (eGFR 30–59 mL/min); 4 = severe CKD (eGFR 15–29 mL/min); and 5 = end-stage CKD (eGFR < 15 mL/min). The progression of renal function was defined as a change in a minimal percentage of decrease in eGFR (25% or greater), as adopted in our previous study [16]. The therapeutic efficacy endpoint was SVR12 (HCV RNA < lower limit of quantification at off-treatment week 12).

## 3. Statistical Analysis

Baseline characteristics including host and viral factors, laboratory data, and underlying comorbidities and pretreatment fibrosis are expressed as means (standard deviation [SD]) and percentages. The proportion of SVR12 achievement is expressed as values and percentages. The change in eGFR during and after SOF/VEL-based therapy was assessed, and trends are shown as figures. Multivariate logistic regression models were used to identify factors including age, gender, presence of cirrhosis, HCC, diabetes mellitus (DM), use of RBV, baseline FIB-4, concomitant hepatitis B virus infection, and history of PEGylated interferon-based antiviral therapy associated with renal function deterioration at EOT, SVR12, SVR24, and SVR48. All analyses were performed using the Statistical Program for Social Sciences (SPSS Statistics Version 23.0, IBM Corp., Armonk, NY, USA).

## 4. Results

### 4.1. Patient Characteristics

The baseline demographic, virological, and clinical characteristics of the 594 patients were included in the per-protocol analysis, and are summarized in Table 1. The mean age was 63.1 years for the total cohort, and male patients accounted for 47.4%. Only 7.1% (*n* = 39) of the cohort were treatment-experienced with IFN-based therapy. Genotype (GT) 2 HCV was the predominant genotype (50%), followed by GT 1b (32.8%). The presence of cirrhosis was seen in 29.3% (*n* = 111/371) of the cohort. The presence of cirrhosis defined by FibroScan was seen in 122 (20.5%) patients, including the known clinically diagnosed cirrhosis of available data (111 patients). Notably, 177 (29.8%) patients were classified in unknown stages of fibrosis because the results of FibroScans were unavailable; all of them were assumed to have non-cirrhosis condition according to their laboratory data, clinical condition, and abdominal ultrasound. Moreover, 65 patients had decompensated cirrhosis, and a total of 68 (12.4%) patients received a combination of SOF/VEL and RBV. Approximately 73 (13.5%) patients had a diagnosis of HCC before the administration of DAA. Forty-five (7.5%) patients were co-infected with HBV. A total of 286 patients treated in June and July of 2019 underwent follow-up at 48 weeks after the end of treatment, including 2 patients with failed viral eradication. There was no liver-related mortality observed.

Twenty-one patients who died during the study period were excluded from per-protocol analysis; the data are summarized in Table 2; their mean age was 68.7 years. Four patients died before the end of treatment, and 17 patients died between end of treatment and off-treatment week 12. Causes of mortality included HCC (*n* = 5), decompensated cirrhosis (*n* = 1), mixed etiology (*n* = 6), and severe esophageal variceal bleeding (*n* = 2). Six out of seven patients with decompensated cirrhosis received SOF/VEL and RBV. One patient did not receive RBV given a history of severe hemolysis. Notably, no patients had advanced-stage CKD before initiation of SOF/VEL treatment. Renal function did not alter the decision to use SOF/VEL, nor modification of dose.

### 4.2. Overall Response to Antiviral Treatment

As shown in Table 3, the overall SVR12 rate was 99.3% (590/594). By HCV genotype, the SVR12 rate was 100% for GT-1, 99.3% for GT-2, 92.9% for GT-3, and 100% for GT-6 patients. The SVR12 rate was 99.3% for treatment-naïve patients, and 100% for patients with previous IFN-based therapy. The SVR12 rate was 100% among patients with HBV and HCV co-infection. The SVR12 rate was comparably high with respect to FIB-4 (99.4%, <3.25 group versus 99.3%, ≥3.25 group). The SVR12 rate was 99.2% among cirrhotic patients. In cirrhotic patients treated with a combination of SOF/VEL and RBV, the SVR12 rate was also higher than in those without RBV (98.5% with RBV versus 99.4% without RBV). There was no significant difference with respect to the SVR12 rates of all subgroups.

### 4.3. Dynamic Changes in ALT, Total Bilirubin, and FIB-4 during the Study Period

**Figure 1:**
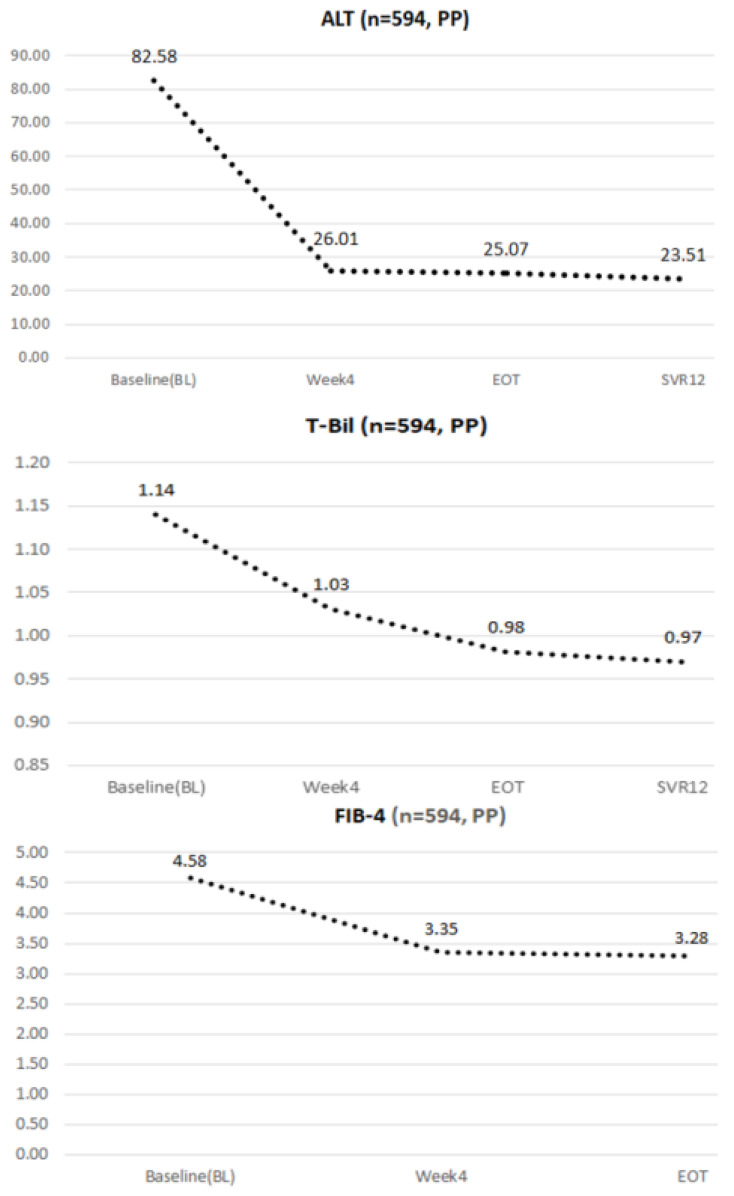
The dynamic changes in laboratory data among enrolled patients who received 12 weeks of SOF/VEL-based therapy.

As shown in Figure 1, HCV-infected patients experienced elevated liver function at baseline, and had markedly decreased ALT after SOF/VEL at on-treatment week 4 and EOT. Patients also saw continuously normal liver function at off-treatment week 12 (82.58, baseline → 26.01, on-treatment week 4 → 25.07, EOT → 23.51, off-treatment week 12; ∆ = −71%). Continuously improving total bilirubin was also observed during the study period (1.14, baseline → 1.03, on-treatment week 4 → 0.98, end of treatment → 0.97, off-treatment week 12; ∆ = −15%). As for fibrosis, patients receiving SOF/VEL therapy showed improved FIB-4 index after completion of treatment (4.58, baseline → 3.35, on-treatment week 4 → 3.28, EOT).

### 4.4. The Dynamic Changes in eGFR during the Study Period

**Figure 2:**
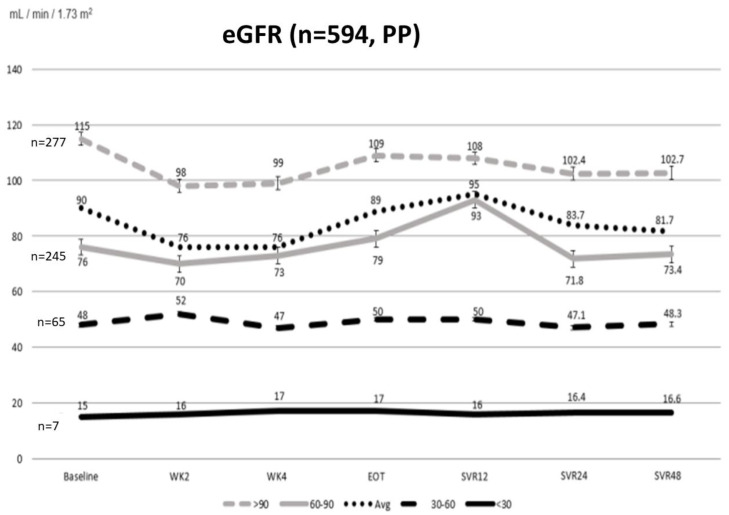
Evolution of eGFR among enrolled patients who received 12 weeks of SOF/VEL-based therapy.

As shown in Figure 2, all patients showed transient on-treatment deterioration of renal function but off-treatment improvement of eGFR from baseline to SVR12. Interestingly, recurrent decline in eGFR was observed from SVR12 to SVR24, and from SVR24 to SVR48. The trend was more significant when classified by stage of chronic kidney disease; those with eGFR ≥ 60 (mL/min/1.73 m^2^) had transient on-treatment deterioration of eGFR, but returned to baseline or improved following SOF/VEL treatment, then developed recurrent decline in eGFR during the study period (baseline versus SVR24 (*n* = 317): 97.71 vs. 88.49, *p* < 0.001; baseline versus SVR48 (*n* = 276): 95.09 vs. 87.47, *p* < 0.001; SVR24 vs. SVR48 (*n* = 222): 88.26 vs. 88.83, *p* = 0.581).

There was no change in eGFR during the study period in patients with baseline eGFR < 60 mL/min/1.73 m^2^. Notably, three patients with CKD stage 4 and four patients with CKD stage 5 did not undergo dialysis but received a full dose of SOF/VEL (400 mg/100 mg tablet once daily). One such patient received SOF/VEL and RBV due to concomitant decompensated liver cirrhosis with ascites. All patients completed treatment, and eGFR remained stable. None of these patients showed symptoms suggesting deterioration of renal function or the need for later dialysis.

### 4.5. Univariate and Multivariate Analysis of Predictive Factors for the Deterioration of Renal Function

The deterioration of renal function was defined as a decrease in eGFR > 25% from baseline to EOT, SVR12, SVR24, and SVR48. The data of all patients are shown in Table 4. The baseline eGFR ≥ 60 (mL/min/1.73 m^2^) was a risk factor for deteriorated renal function at EOT (OR = 2.776, 95% CI: 1.106–6.965, *p* = 0.030) after multivariate analysis. At SVR12, DM (OR = 2.548, 95% CI: 1.093–5.940, *p* = 0.030) and the use of RBV (OR = 4.369, 95% CI: 1.771–10.780, *p* = 0.010) were significant risk factors. At SVR24, the use of RBV, liver cirrhosis, DM, and baseline FIB-4 ≥ 3.25 were risk factors after univariate analysis. After multivariate analysis, DM (OR = 2.702, 95% CI: 1.191–6.131, *p* = 0.017) and baseline FIB-4 ≥ 3.25 (OR = 2.699, 95% CI: 1.050–6.935, *p* = 0.039) were significant risk factors for the deterioration of renal functions. At SVR48, similarly, the use of RBV, liver cirrhosis, diabetes mellitus, and FIB-4 ≥ 3.25 were risk factors for deteriorated renal functions after univariate analysis. After multivariate analysis, diabetes mellitus (OR = 2.572, 95% CI: 1.133–5.836, *p* = 0.024) and the use of RBV (OR = 3.018, 95% CI: 1.156–7.883, *p* = 0.024) were significant risk factors.

We further performed subgroup analysis for patients with baseline eGFR ≥ 60 (mL/min/1.73 m^2^), and the data are shown in Table 5. No specific risk factors for deteriorated renal function were identified at EOT. At SVR12, aging (OR = 4.094, 95% CI: 1.161–14.437, *p* = 0.028) and the use of RBV (OR = 4.671, 95% CI: 1.683–12.960, *p* = 0.003) were significant risk factors. At SVR24, a significant risk of the deterioration of renal function caused by the use of RBV (OR = 5.200, 95% CI: 1.983–13.634, *p* = 0.001) was identified. At SVR48, DM (OR = 2.765, 95% CI: 1.104–6.922, *p* = 0.030) and the use of RBV (OR = 3.143, 95% CI: 1.047–9.435, *p* = 0.041) were identified as significant risk factors for worsening renal function.

## 5. Discussion

SOF/VEL therapy has been covered by Taiwan’s national health insurance system since 1 June 2019. In the present cohort of HCV-infected patients, we observed a high overall SVR12 rate (99.3%) across genotype, past IFN-based treatment, and cirrhosis status—consistent with clinical trials [4,5,6,17,18,19,20,21,22]. Several cohort studies have reported similarly high efficacy rates regardless of patient or viral factors [7,8,9,10,23], with most reported data coming from western countries. In Japan, SOF/VEL treatment is reserved for special groups. Izumi et al. [24] reported 24 weeks of SOF/VEL with RBV to be highly effective and well tolerated in patients who previously failed a DAA-based regimen while suffering from high NS5A-resistance-associated substitutions (RASs). Takehara et al. [25] reported that 12 weeks of SOF/VEL was highly effective for HCV-infected patients with decompensated cirrhosis. To date, information on SOF/VEL treatment is lacking in Asia, making our work the first large cohort study demonstrating that SOF/VEL-based treatment is highly effective for viral eradication.

The potential nephrotoxicity of sofosbuvir-based treatment due to high renal elimination is of concern. Acute kidney injury (AKI) occurs in 1–15% of patients treated with SOF, and may recover following drug discontinuation [26]. Advanced baseline stage of chronic kidney disease is the predominant independent risk factor of eGFR decline when using SOF-based DAAs [14,26]. Therefore, sofosbuvir and/or RBV are not generally recommended for HCV-infected patients with severe renal impairment according to the Asian Pacific Association for the Study of the Liver (APSAL, Tokyo, Japan) guidelines [27]. A recent series of case reports found no safety concerns associated with SOF-based treatment in patients with advanced chronic kidney disease [28]. A pooled meta-analysis by Li et al. also reported that SOF-based treatments are safe for HCV-infected patients with CKD stage 4–5 [29]. Moreover, a phase II single-arm trial by Borgia et al. [15] showed that 12 weeks of SOF/VEL treatment were safe and well tolerated in patients with end-stage renal disease undergoing dialysis.

The renal safety concerns of SOF/VEL treatment in HCV-infected patients are important, but are seldom discussed in western studies [8,9,18]. Meanwhile, our study is the first large-cohort study in Asia to demonstrate long-term follow-up of renal function after SOF/VEL treatment. In this current study, we observed that patients experienced transient on-treatment reduction in renal function that improved upon ending treatment. The eGFR degradation was reversed after drug discontinuation in patients with normal renal function or early-stage chronic kidney disease (CKD stage 1–2). However, those patients experienced recurrent eGFR degradation during one-year follow-up. Aged patients with concomitant use of RBV had significantly higher risk of worsening renal function at SVR12. As time went by, the use of RBV remained a significant risk factor for deteriorated renal function at SVR24. At SVR48, those patients with DM and the use of RBV had significantly higher risk of worsening renal function. The actual mechanism whereby the use of RBV would worsen the renal function was not identified. However, considering that the use of RBV did not alter the SVR rate (with RBV versus without RBV: 98.1% versus 99.3%), further study should be conducted in order to weigh the risks and benefits of RBV in patients with eGFR ≥ 60 (mL/min/1.73 m^2^).

In Taiwan, most clinicians adhere to the APASL clinical practice recommendations, and use glecaprevir/pibrentasvir or elbasvir/grazoprevir for the treatment of HCV-infected patients with advanced CKD. In November 2019, Taiwan’s FDA allowed use of sofosbuvir-containing treatments in patients with an eGFR ≤ 30 mL/min, as well as in those on dialysis. In this study, seven patients with CKD 4 or CKD 5 received SOF/VEL-based treatment after December 2019. One patient received SOF/VEL and RBV due to concomitant decompensated liver cirrhosis with ascites. There was no significant change in eGFR during SOF/VEL treatment and one-year follow-up. None of the patients with CKD stage 4–5 had renal deterioration or progressed to dialysis. The SOF/VEL regimen is quite safe with respect to renal safety in patients with advanced chronic kidney disease in real-world experience.

Our study includes a number of limitations that warrant mentioning: First, we assumed that patients with SOF/VEL and RBV treatment were diagnosed with decompensated cirrhosis before enrollment; however, we found that three patients had no relevant history or laboratory data supporting such a diagnosis—two patients had positive cryoglobulinemia, and the other had no recorded etiology. The SVR12 rate was still high despite this discrepancy. Second, a relatively higher withdrawal rate was observed due to mortality (*n* = 21) during the study period, which was attributed to higher comorbidity and worse clinical condition before SOF/VEL treatment. Of the mortality cases, four patients did not complete the SOF/VEL treatment. Causes of mortality included progression of HCC, sepsis, progression of decompensated cirrhosis, and esophageal variceal bleeding. Third, we aimed to show the real-world data of SOF/VEL treatment in Taiwan and discuss the renal safety even one year after completion of treatment. Inevitably, some patients experienced concomitant HBV and HCC, and received medications affecting their renal functions, such as nucleotide analogue (NUC). We reviewed the patients’ charts, and only seven patients (1.1%) received NUC for HBV during the study period. Finally, the rate of genotype 3 was only 2.4% in this study; this proportion may not reflect global infection distribution.

In conclusion, our study shows that 12 weeks of SOF/VEL therapy achieve high SVR12 rates. For patients with eGFR ≥ 60 (mL/min/1.73 m^2^), recurrent degradation of eGFR was observed at SVR24 and even SVR48—especially in those with diabetes mellitus and the use of RBV. Close monitoring of renal function is warranted.

## Figures and Tables

**Table 1:** Patient details at time of enrollment (*n* = 594, PP *).

Factors	Mean (Range)
Mean age, years (range)	63.1 (19–95)
Male gender, *n* (%)	282 (47.4%)
HCV RNA Q	2,659,919 (=Log 6.4)
HCV genotype, *n* (%)	
GT 1	226 (38.1%)
1a	30 (5.1%)
1b	195 (32.8%)
GT 2	297 (50.0%)
GT 3	14 (2.4%)
GT 6	29 (4.9%)
Mixed/unknown	28 (4.7%)
DM, *n* (%)	78/339 (23.1%)
SOF/VEL + RBV, *n* (%)	68 (12.4%)
Fibrosis stage, *n* (%)	
Non-cirrhosis	295 (49.7%)
Cirrhosis	122 (20.5%)
Unknown	177 (29.8%)
Cirrhosis registered	111/371 (29.3%)
Compensated	46 (12.3%)
Decompensated	65 (17.5%)
Treatment history, *n* (%)	
PEG-IFN experienced	39 (7.1%)
HBV co-infection	45 (7.5%)
HCC, *n* (%)	73 (13.5%)
ALT (U/L)	82.8 (8–2615)
AST (U/L)	74.0 (13–2150)
eGFR (mL/min/1.73 m^2^)	89.9 (6–256)
CKD stage 1	277 (46.6%)
CKD stage 2	245 (41.3%)
CKD stage 3	65 (10.9%)
CKD stage 4	3 (0.5%)
CKD stage 5	4 (0.7%)
Total bilirubin (mg/dL)	1.1 (0.2–27.2)
FIB-4, *n* (%)	
<3.25	316 (53.2%)
≥3.25	275 (46.3%)
Unknown	3 (0.5%)

HCV: hepatitis C virus; DM: diabetes mellitus; RBV: ribavirin; HBV: hepatitis B virus; HCC: hepatocellular carcinoma; ALT: alanine aminotransferase; AST: aspartate transaminase; eGFR: estimated glomerular filtration rate; * PP: Including all patients who received 12 weeks of Epclusa^®^ and HCV RNA data available at post-treatment week 12, and excluding non-virological failures.

**Table 2:** Mortality details.

	Total (*n* = 21)
Mean age, years (range)	68.7 (52–85)
Expire date	
Before EOT	4
Between EOT to off-treatment week 12	17
HCV genotype, *n* (%)	
GT 1b	4 (19.0%)
GT 2	16 (76.2%)
GT 6	1 (4.8%)
SOF/VEL + RBV, *n* (%)	6 (28.5%) ^a^
Causes of mortality	
HCC	5 (23.9%)
Decompensated cirrhosis	1 (4.8%)
Mixed HCC/decompensated cirrhosis	6 (28.5%)
EVB	2 (9.5%)
Others/unknown	7 (33.3%) ^b^
Cirrhosis registered	14 (66.7%)
ALT (U/L)	116.7 (17–789)
AST (U/L)	197.8 (29–407)
eGFR (mL/min/1.73 m^2^)	75.64 (37–166)
CKD Stage 1	5 (23.9%)
CKD Stage 2	10 (47.6%)
CKD Stage 3	6 (28.5%)
Total bilirubin (mg/dL)	5.8 (0.5–14.1)

EOT: end of treatment; HCV: hepatitis C virus; RBV: ribavirin; HCC: hepatocellular carcinoma; EVB: esophageal variceal bleeding; ALT: alanine aminotransferase; AST: aspartate transaminase; eGFR: estimated glomerular filtration rate; CKD: chronic kidney disease. ^a^: One patient did not receive ribavirin due to history of severe hemolysis. ^b^: Sepsis, cholangiocarcinoma with portal vein thrombosis and lymph node metastases, cervical cancer, and 4 unknown etiologies.

**Table 3:** SVR12 results for different subgroups in CGMH RWD.

Factors		SVR12 (*n* = 594 by PP *)	
Overall		590/594	(99.3%)
Genotype	1a	30/30	(100%)
	1b	195/195	(100%)
	2	295/297	(99.3%)
	3	13/14	(92.9%)
	6	29/29	(100%)
	Mixed	27/28	(96.4%)
Peg-IFN experienced	Naïve	548/552	(99.3%)
	Experienced	41/41	(100%)
HBV/HCV co-infection	HBV (+)	45/45	(100%)
	HBV (−)	540/544	(99.3%)
FIB-4	<3.25	314/316	(99.4%)
	≥3.25	273/275	(99.3%)
Cirrhosis	Liver cirrhosis	121/122	(99.2%)
	Non-liver cirrhosis	292/295	(99.0%)
	Unknown	177/177	(100%)
+RBV	With RBV	67/68	(98.5%)
	Without RBV	523/526	(99.4%)

HBV: hepatitis B virus; HCV: hepatitis C virus; RBV: ribavirin; * PP: Including all patients who received 12 weeks of Epclusa^®^ and HCV RNA data available at post-treatment week 12, excluding non-virological failures. All subgroups showed non-significant *p*-values.

**Table 4:** Univariate and multivariate analysis of risk factors for deteriorated renal function of all patients.

EOT		Univariate		Multivariate	
Variable	Comparison	OR (95% CI)	*p*-Value	OR (95% CI)	*p*-Value
Age (years)	≥60 vs. <60	0.715 (0.419–1.221)	0.219		
Sex	M vs. F	0.610 (0.352–1.057)	0.078		
Liver cirrhosis	Yes vs. No	1.262 (0.734–2.172)	0.400		
HCC	Yes vs. No	1.447 (0.737–2.843)	0.282		
Diabetes mellitus	Yes vs. No	1.508 (0.641–3.548)	0.347		
Ribavirin	Yes vs. No	1.373 (0.628–3.000)	0.427		
Baseline eGFR	≥60 vs. <60	1.508 (0.731–3.111)	0.266	2.776 (1.106–6.965)	0.030
Base_FIB-4	≥3.25 vs. <3.25	1.077 (0.632–1.835)	0.786		
HBV	Yes vs. No	0.681 (0.232–1.999)	0.485		
History of PR use	Yes vs. No	0.866 (0.324–2.315)	0.774		
**SVR12**		**Univariate**		**Multivariate**	
Age (years)	≥60 vs. <60	1.366 (0.745–2.504)	0.314		
Sex	M vs. F	1.526 (0.859–2.712)	0.149		
Liver cirrhosis	Yes vs. No	2.130 (1.199–3.786)	0.010		
HCC	Yes vs. No	1.637 (0.783–3.423)	0.190		
Diabetes mellitus	Yes vs. No	3.009 (1.376–6.578)	0.006	2.548 (1.093–5.940)	0.030
Ribavirin	Yes vs. No	3.681 (1.889–7.174)	<0.001	4.369 (1.771–10.78)	0.010
Baseline eGFR	≥60 vs. <60	1.527 (0.682–3.419)	0.304		
Base_FIB-4	≥3.25 vs. <3.25	1.245 (0.696–2.228)	0.460		
HBV	Yes vs. No	0.429 (0.101–1.824)	0.252		
History of PR use	Yes vs. No	0.773 (0.230–2.599)	0.677		
**SVR24**		**Univariate**		**Multivariate**	
Age (years)	≥60 vs. <60	1.026 (0.568–1.852)	0.933		
Sex	M vs. F	1.492 (0.845–2.633)	0.168		
Liver cirrhosis	Yes vs. No	2.753 (1.540–4.920)	0.001		
HCC	Yes vs. No	1.679 (0.856–3.293)	0.132		
Diabetes mellitus	Yes vs. No	2.500 (1.148–5.445)	0.021	2.702 (1.191–6.131)	0.017
Ribavirin	Yes vs. No	3.632 (1.902–6.934)	<0.001	2.428 (0.981–6.006)	0.055
Baseline eGFR	≥60 vs. <60	1.150 (0.490–2.702)	0.748		
Base_FIB-4	≥3.25 vs. <3.25	2.124 (1.150–3.922)	0.016	2.699 (1.050–6.935)	0.039
HBV	Yes vs. No	0.952 (0.316–2.863)	0.930		
History of PR use	Yes vs. No	0.507 (0.150–1.718)	0.276		
**SVR48**		**Univariate**		**Multivariate**	
Age (years)	≥60 vs. <60	1.298 (0.666–2.532)	0.444		
Sex	M vs. F	1.250 (0.680–2.297)	0.472		
Liver cirrhosis	Yes vs. No	2.192 (1.184–4.059)	0.013		
HCC	Yes vs. No	0.265 (0.043–1.636)	0.153		
Diabetes mellitus	Yes vs. No	2.524 (1.129–5.639)	0.024	2.572 (1.133–5.836)	0.024
Ribavirin	Yes vs. No	2.560 (1.235–5.305)	0.011	3.018 (1.156–7.883)	0.024
Baseline eGFR	≥60 vs. <60	1.000 (0.419–2.385)	1.000		
Base_FIB-4	≥3.25 vs. <3.25	1.910 (1.013–3.601)	0.045		
HBV	Yes vs. No	1.622 (0.573–4.596)	0.362		
History of PR use	Yes vs. No	1.133 (0.370–3.471)	0.827		

Definition of progression in renal function: >25% decrease in eGFR from baseline to EOT, SVR24, or SVR48. Abbreviations—HCC: hepatocellular carcinoma; eGFR: estimated glomerular filtration rate; HBV: hepatitis B virus; PR: PEGylated interferon and ribavirin.

**Table 5:** Univariate and multivariate analysis of risk factors for the deterioration of renal function (eGFR > 60 mL/min/1.73 m^2^).

EOT		Univariate		Multivariate	
Variable	Comparison	OR (95%CI)	*p*-Value	OR (95% CI)	*p*-Value
Age (years)	≥60 vs. <60	0.661 (0.371–1.180)	0.162		
Sex	M vs. F	0.594 (0.325–1.082)	0.089		
Liver cirrhosis	Yes vs. No	1.180 (0.651–2.138)	0.585		
HCC	Yes vs. No	1.432 (0.671–3.057)	0.353		
Diabetes mellitus	Yes vs. No	1.167 (0.400–3.406)	0.778		
Ribavirin	Yes vs. No	1.102 (0.437–2.775)	0.837		
Base_FIB-4	≥3.25 vs. <3.25	1.149 (0.641–2.059)	0.640		
HBV	Yes vs. No	0.601 (0.176–2.050)	0.416		
History of PR use	Yes vs. No	1.003 (0.370–2.716)	0.996		
**SVR12**		**Univariate**		**Multivariate**	
Age (years)	≥60 vs. <60	1.895 (0.943–3.809)	0.073	4.094 (1.161–14.437)	0.028
Sex	M vs. F	1.361 (0.717–2.585)	0.346		
Liver cirrhosis	Yes vs. No	2.082 (1.093–3.964)	0.026		
HCC	Yes vs. No	2.178 (0.984–4.823)	0.055		
Diabetes mellitus	Yes vs. No	1.599 (0.595–4.298)	0.352		
Ribavirin	Yes vs. No	4.200 (1.990–8.865)	<0.001	4.671 (1.683–12.960)	0.003
Base_FIB-4	≥3.25 vs. <3.25	1.628 (0.846–3.130)	0.144		
HBV	Yes vs. No	0.535 (0.124–2.301)	0.401		
History of PR use	Yes vs. No	0.927 (0.272–3.156)	0.904		
**SVR24**		**Univariate**		**Multivariate**	
Age (years)	≥60 vs. <60	1.152 (0.615–2.158)	0.658		
Sex	M vs. F	1.468 (0.799–2.696)	0.216		
Liver cirrhosis	Yes vs. No	2.761 (1.487–5.127)	0.001		
HCC	Yes vs. No	1.792 (0.863–3.722)	0.118		
Diabetes mellitus	Yes vs. No	2.292 (0.956–5.492)	0.063		
Ribavirin	Yes vs. No	5.214 (2.576–10.553)	<0.001	5.200 (1.983–13.634)	0.001
Base_FIB-4	≥3.25 vs. <3.25	2.088 (1.083–4.022)	0.028		
HBV	Yes vs. No	1.074 (0.351–3.287)	0.901		
History of PR use	Yes vs. No	0.524 (0.153–1.791)	0.303		
**SVR48**		**Univariate**		**Multivariate**	
Age (years)	≥60 vs. <60	1.649 (0.804–3.384)	0.173		
Sex	M vs. F	1.147 (0.594–2.212)	0.683		
Liver cirrhosis	Yes vs. No	1.792 (0.926–3.469)	0.083		
HCC	Yes vs. No	0.785 (0.310–1.984)	0.608		
Diabetes mellitus	Yes vs. No	2.621 (1.068–6.433)	0.035	2.765 (1.104–6.922)	0.030
Ribavirin	Yes vs. No	2.396 (1.060–5.415)	0.036	3.143 (1.047–9.435)	0.041
Base_FIB-4	≥3.25 vs. <3.25	1.433 (0.735–2.794)	0.291		
HBV	Yes vs. No	2.124 (0.722–6.246)	0.171		
History of PR use	Yes vs. No	1.191 (0.384–3.695)	0.762		

Definition of progression in renal function: >25% decrease in eGFR from baseline to EOT, SVR24, or SVR48. Abbreviations—HCC: hepatocellular carcinoma; eGFR: estimated glomerular filtration rate; HBV: hepatitis B virus; PR: PEGylated interferon and ribavirin.
